# Unusual Presentation of Synovial Lipomatosis Mimicking As Septic Arthritis of Knee: A Case Report

**DOI:** 10.7759/cureus.58075

**Published:** 2024-04-11

**Authors:** Krishnavel Thavasianantham, TS Raagul, A Ganesh, Pradeep Elangovan, Haemanath P, Pooja S Regunathan

**Affiliations:** 1 Orthopaedics and Traumatology, Chettinad Hospital and Research Institute, Chennai, IND; 2 Orthopaedics, Institute of Medical Sciences and SUM Hospital, Bhubaneswar, IND

**Keywords:** knee pain, arthrotomy, synovial lipomatosis, septic arthritis, knee

## Abstract

Synovial lipomatosis or lipoma arborescens is a very uncommon pseudo-tumorous lesion of the synovium which more commonly affects the knee joint. The most probable cause of this pathological lesion is degenerative articular disorders of the joint and improper fat accumulation. It is characterized by presence of villous proliferation of the synovium and replacement of the sub-synovial tissue by mature adipocytes which is infiltrated by dense chronic inflammatory cells like lymphocytes, plasma cells and eosinophils. This condition is rarely seen in smaller joints. Its aetiology is still unknown. We report a patient who presented with features of septic arthritis which on intraoperative and histopathological assessment showed features of synovial lipomatosis.

## Introduction

Synovial lipomatosis of the knee, also known as Hoffa's disease or villonodular synovitis with lipomatosis, is a rare but intriguing condition within the realm of orthopaedics [1]. According to Miladore et al., this disorder involves the proliferation of fatty tissue within the synovial lining of the knee joint, leading to a spectrum of symptoms ranging from mild discomfort to significant functional impairment [2]. While relatively uncommon, the understanding and management of synovial lipomatosis is of paramount importance for orthopaedic surgeons, as it poses diagnostic challenges and necessitates tailored treatment strategies [3].

It can affect any age starting from eight years to 80 years, but mostly it is reported among middle-aged males. The patient usually complains of pain and swelling of the affected joint. It appears to be more common in the knee, although it has also been recorded in the wrist and ankle, suggesting that it may be less common in smaller joints [4].

Complete synovectomy, either by open debridement or arthroscopically, is the advised course of treatment. However, when it comes to the question of the best method, it is always put into debate as both have shown positive results despite the paucity of follow-up outcome data [5]. This case report provides insight into the new way of presentation of synovial lipomatosis. 

## Case presentation

A 20-year-old male came to the outpatient department with complaints of pain and swelling over his right knee for the past seven days. The patient gave a history of a low-velocity road traffic accident around 20 days back following which the patient had a history of fever for the past four days. The patient also gave a history of difficulty in weight-bearing and walking using the right lower limb.

**Figure 1:**
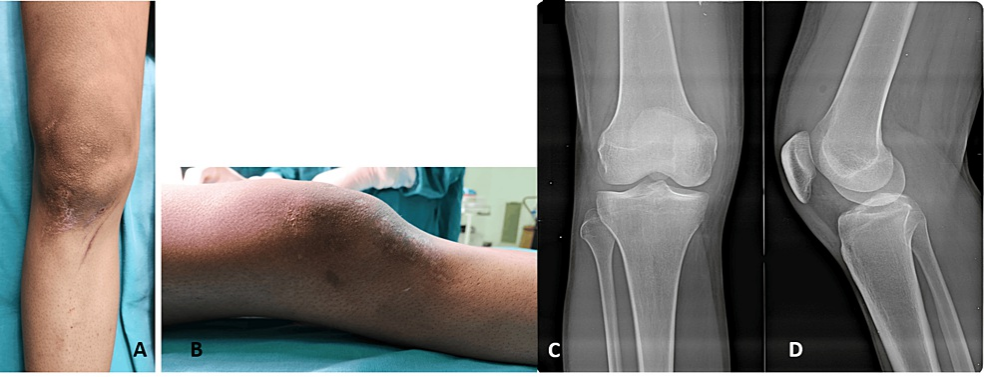
Clinical pictures and X-rays at the time of presentation. Clinical pictures (A & B) showing moderate effusion of the right knee. Antero-posterior view (C) and lateral view (D) of radiograph showing no obvious bony injury.

On examination of his right knee showed large effusion with local warmth and diffuse tenderness. Supra-patellar fullness was present and the patellar tap test was positive (Figure 1A, 1B). The range of motion across the right knee was from 0 degrees to 50 degrees and further movement was restricted due to pain. The patient did not have any distal neurovascular deficit. The radiograph of the right knee showed no obvious bony abnormality (Figure 1C, 1D).

**Figure 2:**
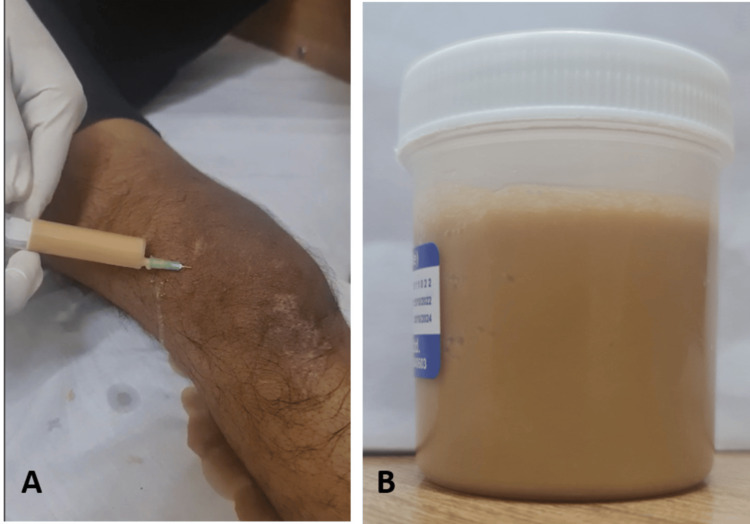
Arthrocentesis of the right knee. Clinical picture (A) showing the arthrocentesis performed. Picture (B) showing aspirated fluid from right knee.

Arthrocentesis of the right knee was done and pus-like fluid was aspirated (Figure 2).

**Figure 3:**
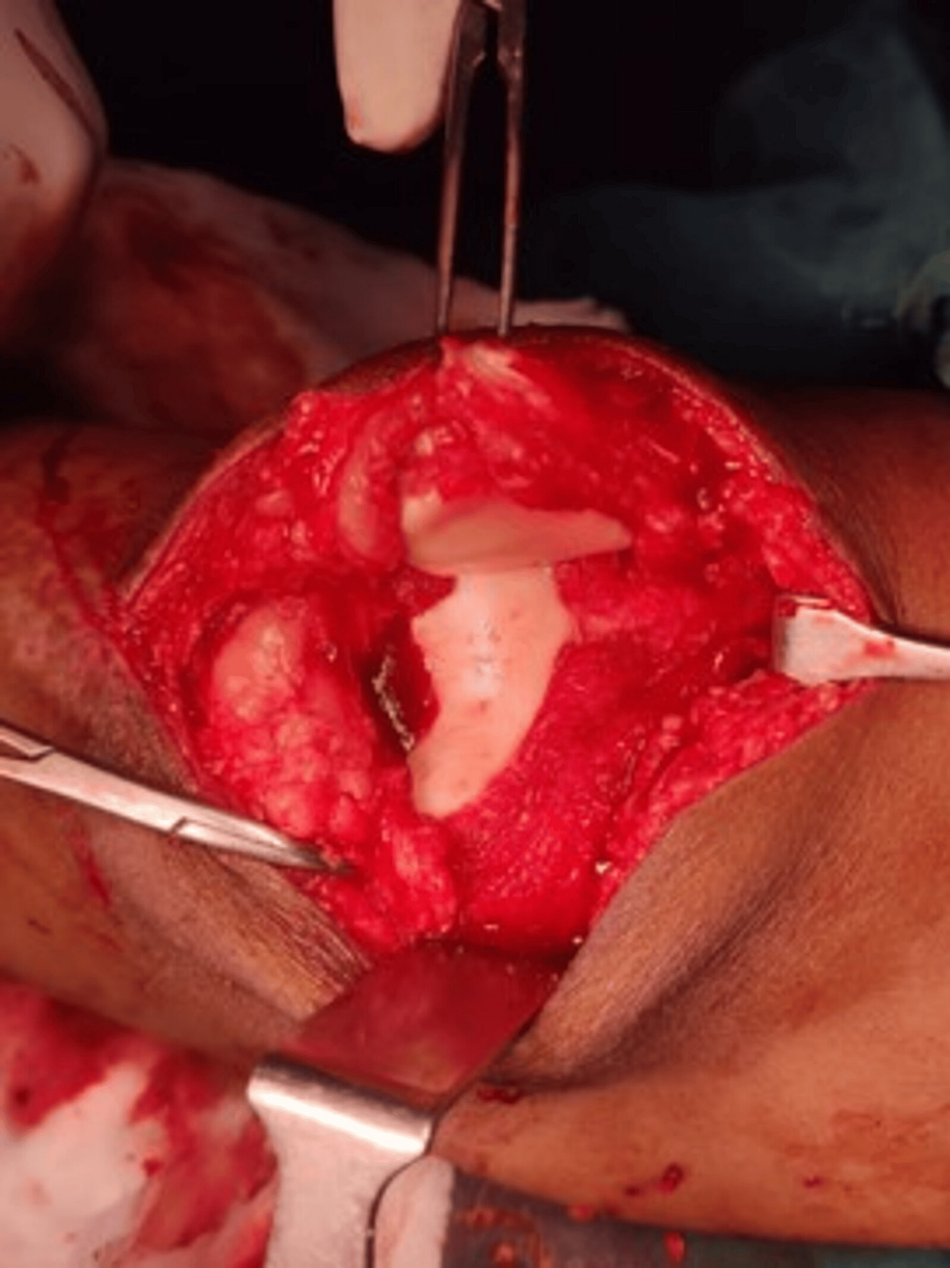
Intra-operative image. It shows fat-like deposits in the articular surface and synovium.

Synovial fluid was sent for Gram staining, acid-fast bacteria (AFB) staining, and culture sensitivity. The patient was provisionally diagnosed with septic arthritis of the knee and was taken up for emergency arthrotomy. Intra-operatively, pus-like fluid was present along with fat-like deposits that were found adherent to the synovium and the articular surface (Figure 3). A partial synovectomy was done and synovial tissue was sent for culture sensitivity, Gram staining, Ziehl-Nelson staining, and histopathological examination.

**Figure 4:**
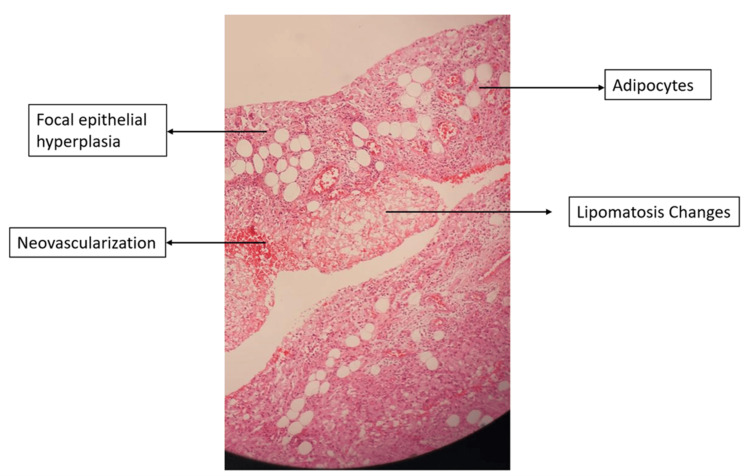
Histopathological slide image of the synovial biopsy (hematoxylin-eosin stain, original magnification × 10).

Pre-operative and intra-operative staining came back negative and the culture report showed no growth. The histopathological report showed focal hyperplasia of synovial epithelium, subepithelial collection of foamy histocytes, abundant mature adipose tissue with an area of fibrosis, congested blood vessels and focal fat necrosis which was suggestive of synovial lipomatosis (Figure 4).

The patient had considerable improvement in pain and effusion. The patient had serial follow-ups at two, four, six, and eight weeks. Knee range of motion had improved periodically and the patient was able to walk pain-free at the end of six weeks. Inflammatory markers were reassessed at the end of the eighth week which showed decreasing trend.

## Discussion

The first documented case of synovial lipomatosis was reported by Hoffa in 1904, who described the condition as "giant synovial osteochondromatosis." Since then, advancements in imaging modalities such as magnetic resonance imaging (MRI) have improved our understanding and diagnostic capabilities for this condition [6]. According to Burguêz et al., this condition is characterized by the abnormal deposition of mature adipose tissue which is interspersed within the synovial membrane, that results in synovial hyperplasia and inflammation [7].

The etiology of synovial lipomatosis remains poorly understood, with various hypotheses implicating chronic inflammation, trauma, mechanical stress, and metabolic factors in its pathogenesis. While it often presents as an isolated phenomenon, synovial lipomatosis has been associated with underlying joint pathology, including osteoarthritis, rheumatoid arthritis, and pigmented villonodular synovitis (PVNS). Furthermore, rare cases of neoplastic transformation within the lipomatous tissue have been reported, underscoring the importance of thorough evaluation and histopathological analysis [8].

Diagnostic evaluation of synovial lipomatosis typically involves a combination of clinical assessment, imaging studies, and arthroscopic evaluation. Magnetic resonance imaging (MRI) is particularly valuable in delineating the extent of synovial proliferation and characterizing the adipose content within the joint space. Arthroscopy allows for direct visualization and biopsy of the synovial tissue, facilitating both diagnosis and therapeutic intervention [9].

According to Kamath et al., management strategies for synovial lipomatosis of the knee encompass a spectrum of conservative and surgical approaches, depending on the severity of symptoms and the extent of joint involvement [5]. Conservative measures such as activity modification, physical therapy, and nonsteroidal anti-inflammatory drugs (NSAIDs) may provide symptomatic relief in milder cases. However, surgical intervention, including arthroscopic synovectomy or open excision of lipomatous tissue, is often required to alleviate pain, restore function, and prevent disease progression.

To differentiate synovial lipomatosis from other neoplastic and non-neoplastic lesions and identify the best course of treatment and prognosis, it is important to take this unusual condition into account while assessing lesions surrounding the knee joint that have either an acute or chronic appearance.

Degenerative articular disorders of the joint and improper fat accumulation may be the cause of synovial lipomatosis, a pseudotumorous lesion of the synovium with a characteristic histomorphology.

In our case scenario, the diagnosis was based on the presenting features of the patient along with the presence of pus-like exudate which was aspirated during knee arthrocentesis. Taking all these factors into account we suspected septic arthritis which on histopathological examination turned out to be synovial lipomatosis.

## Conclusions

In conclusion, synovial lipomatosis of the knee poses diagnostic challenges and necessitates a multidisciplinary approach to management. Advancements in imaging modalities and histopathological analysis have improved our understanding of this condition, enabling more precise diagnosis and targeted therapeutic interventions. Further research is warranted to elucidate the underlying pathophysiological mechanisms and optimize treatment strategies for this intriguing orthopaedic entity.

With this study, we emphasize making a practice of taking a synovial biopsy in all suspected cases of septic arthritis, as you may be surprised to have a case like this.
